# Structural Color Filters Enabled by a Dielectric Metasurface Incorporating Hydrogenated Amorphous Silicon Nanodisks

**DOI:** 10.1038/s41598-017-02911-w

**Published:** 2017-05-31

**Authors:** Chul-Soon Park, Vivek Raj Shrestha, Wenjing Yue, Song Gao, Sang-Shin Lee, Eun-Soo Kim, Duk-Yong Choi

**Affiliations:** 1 Department of Electronic Engineering, Kwangwoon University, 20 Kwangwoon-ro, Nowon-gu, Seoul, 01897 South Korea; 2 School of Physics, The University of Melbourne, Melbourne, Victoria, 3010 Australia; 3 Laser Physics Centre, Research School of Physics and Engineering, Australian National University, Canberra, ACT 2601 Australia

## Abstract

It is advantageous to construct a dielectric metasurface in silicon due to its compatibility with cost-effective, mature processes for complementary metal-oxide-semiconductor devices. However, high-quality crystalline-silicon films are difficult to grow on foreign substrates. In this work, we propose and realize highly efficient structural color filters based on a dielectric metasurface exploiting hydrogenated amorphous silicon (a-Si:H), known to be lossy in the visible regime. The metasurface is comprised of an array of a-Si:H nanodisks embedded in a polymer, providing a homogeneously planarized surface that is crucial for practical applications. The a-Si:H nanodisk element is deemed to individually support an electric dipole (ED) and magnetic dipole (MD) resonance via Mie scattering, thereby leading to wavelength-dependent filtering characteristics. The ED and MD can be precisely identified by observing the resonant field profiles with the assistance of finite-difference time-domain simulations. The completed color filters provide a high transmission of around 90% in the off-resonance band longer than their resonant wavelengths, exhibiting vivid subtractive colors. A wide range of colors can be facilitated by tuning the resonance by adjusting the structural parameters like the period and diameter of the a-Si:H nanodisk. The proposed devices will be actively utilized to implement color displays, imaging devices, and photorealistic color printing.

## Introduction

Metasurface alludes to an ultra-thin optical planar structure that comprises a two-dimensional (2D) arrangement of nanoscale scatterers, mimicking the functionalities of an artificial metamaterial^1, 2^. Recently, various types of metasurfaces were extensively researched due to their outstanding capabilities to deal with the polarization, phase, and amplitude of light, potentially serving as a miniaturized version of the conventional free-space optics components that include lenses, waveplates, and spectral filters^1–4^. Metasurfaces based on a metal-dielectric plasmonic nanostructure inevitably suffered from a significant absorption caused by the metal^5, 6^. To mitigate such metallic loss, an all-dielectric metasurface resorting to high-index dielectric materials such as silicon (Si) that relies on an electric dipole (ED) and a magnetic dipole (MD), which are mediated by the Mie scattering, was proposed as a prime alternative to a plasmonic metasurface^7–11^.

One of the outstanding applications of such a metasurface may be a structural color filter that operates in the visible regime, which is regarded as a promising alternative for pigment/dye-based colorations in the applications encompassing color displays, imaging, color printing, photovoltaic, and so forth^12–15^. To date, diverse structural filters were demonstrated utilizing metal-dielectric nanostructures like a plasmonic and Fabry-Perot resonator^16–23^. They are however prone to a severe loss that is usually incurred by the metallic layer in the visible regime. Lately, plasmonic color filters enabling a high efficiency have been reported^22–26^. However, the inherent loss of metals associated with the filters might possibly hinder the flexible control of their relatively broadened bandwidth. To the contrary, an all-dielectric structural filter was intensively attempted based on crystalline silicon (c-Si) with a view to a multi-color generation^27–35^. For the c-Si based colorations, the transmission was unsatisfactory while the growing of a high-quality c-Si film on a foreign substrate remains a major challenge yet. In fact, processes like a laser/thermal annealing and an atomic layer deposition were additionally introduced to form a c-Si layer on a glass substrate^36, 37^. A color filter that taps into c-Si nanowires of a high aspect ratio was also studied to improve the performance to a certain extent^30–32^. Noting that the diameter and height are typically in the range of tens of nanometers and several micrometers, respectively, the device might be susceptible to deteriorated fragility while additional metal masks are demanded for its fabrication^30, 31^. In the case of the color filter that exploits Si nanodisks of a low aspect ratio, the efficiency was unacceptably low^35, 38^. As against the case of c-Si, amorphous silicon (a-Si) is presumed to offer a salient advantage that it can be efficiently grown over a foreign substrate at a low temperature so as to exhibit a high refractive index and be appreciably compatible with the cost-effective complementary metal-oxide-semiconductor (CMOS) process. In this context, several color filtering schemes were suggested relying on either a high-index film or a nanodisk in a-Si, yet the transmission was unbearably poor or the operation was limited to the reflective mode due to its high absorption in the visible band^20, 21, 39, 40^. To the best of our knowledge, there has been no report on a highly transmissive structural color filter that capitalizes on a subwavelength a-Si nanodisk, whose operation is valid throughout the visible band. In this paper, we embody a dielectric metasurface serving as a highly efficient subtractive color filter. The metasurface resorts to an ultra-thin nanodisk in hydrogenated amorphous silicon (a-Si:H), which can be grown at a low temperature to provide superior optical properties in conjunction with a high index. A variety of colors were obtained via tailoring of the structural parameters associated with the a-Si:H nanodisk. The operation of the proposed metasurface was thoroughly explored through the observation of the field profiles, which are accountable for the ED and MD resonances initiated by the a-Si:H nanodisk.

## Results

### Subtractive color filters exploiting an a-Si:H metasurface

**Figure 1:**
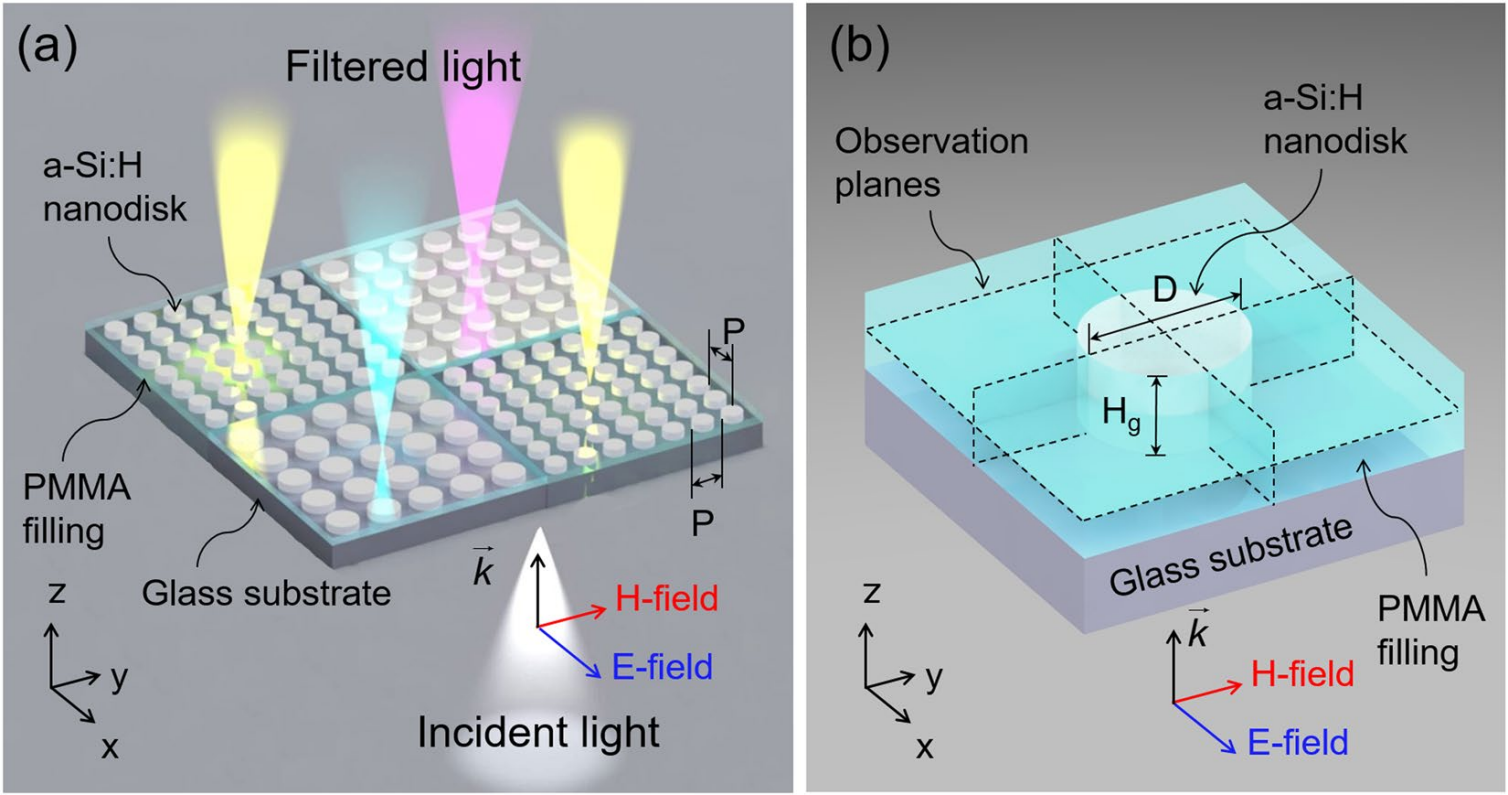
Configuration of the proposed structural color filters resorting to an a-Si:H metasurface. (**a**) Each filter consists of a 2D lattice of a-Si:H nanodisks on a glass substrate. The nanodisk is embedded in a polymeric material of PMMA, which serves as an index-matching layer. The incident white light is filtered into distinct visible colors depending on the period P and the diameter D. (**b**) Schematic of a unit a-Si:H nanodisk, with the observation planes defined by the dotted line.

Figure 1 shows a schematic of the proposed structural color filter that draws upon a dielectric metasurface. As depicted in Fig. 1(a), the metasurface incorporates a 2D array of a-Si:H nanodisks on a glass substrate. The neighborhood of each nanodisk is filled with a polymer of poly-methyl methacrylate (PMMA), with a refractive index of 1.49, which is close to that of the substrate. The polymeric material acts as an index-matching layer, thereby establishing a homogeneous optical environment from the viewpoint of the nanodisk^7, 8^. An enlarged view of the elemental nanodisk is depicted in Fig. 1(b). The polarization for incident light is indicated by the alignment of the electric field (E-field) with respect to the x-direction. The incoming light can be filtered into distinct colors in accordance with the structural parameters in relation to the nanodisk, encompassing the period (P), diameter (D), and height (H_g_). The proposed filters were designed by means of a simulation tool that is based on the finite-difference time-domain (FDTD) method (FDTD Solutions, Lumerical, Canada). For an a-Si:H film that is deposited via the chemical vapor deposition (CVD) at a low temperature of 250 °C, the refractive index was practically measured and plotted in supplementary Fig. S1.

**Figure 2:**
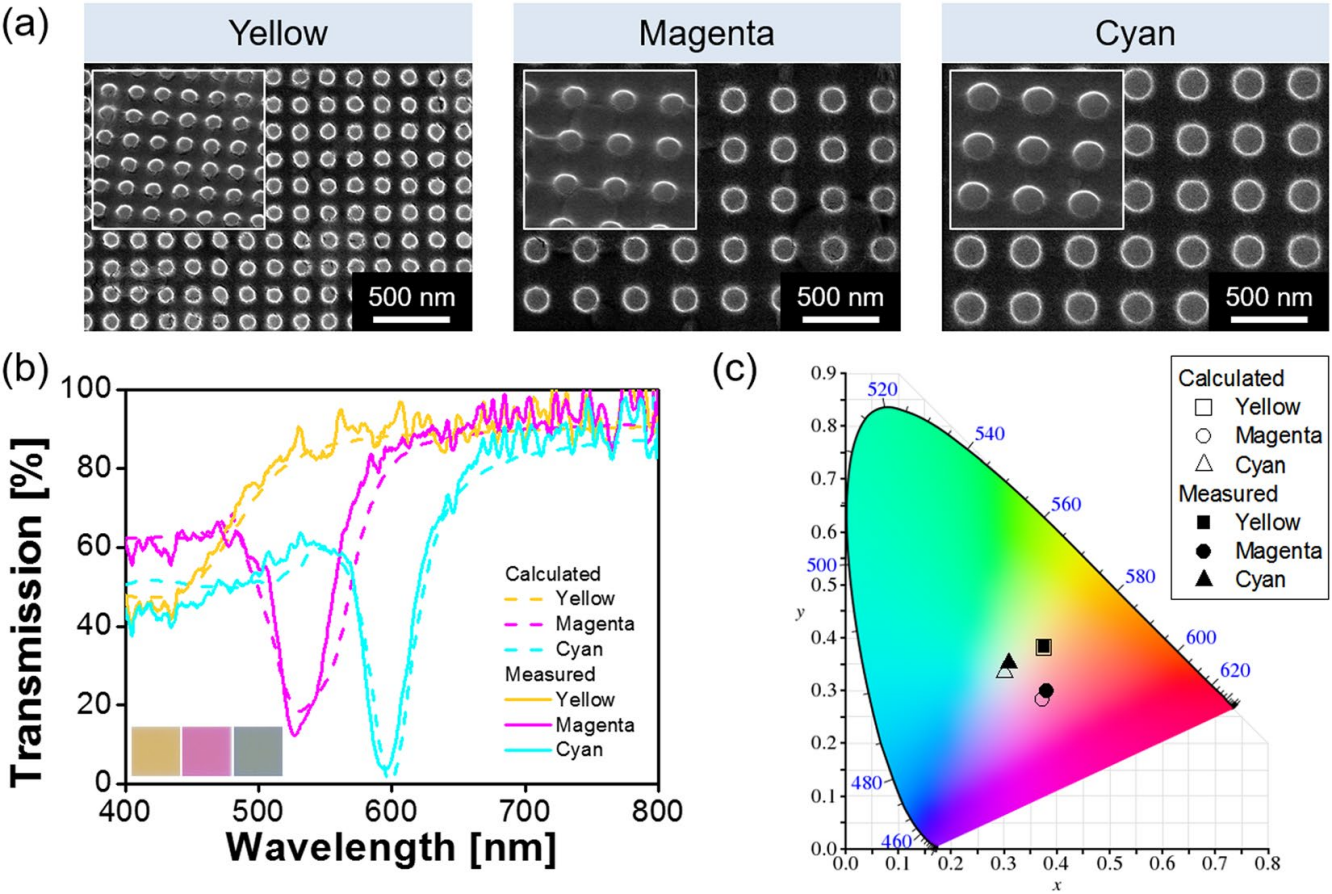
(**a**) SEM images of the fabricated metasurfaces for yellow, magenta, and cyan colors, from left to right. The scale bar is equivalent to 500 nm. (**b**) Calculated (dashed) and measured (solid) transmission characteristics for the three primary subtractive colors of yellow, magenta, and cyan. The inset shows the generated color output for a single pixel. (**c**) The corresponding color outputs are mapped in the CIE 1931 chromaticity diagram.

With the intention of generating the three primary subtractive colors of yellow, magenta, and cyan, the a-Si:H nanodisk was devised to have periods of P = 150 nm, 330 nm, and 370 nm, and diameters of D = 80 nm, 130 nm, and 170 nm, respectively. The height of the nanodisk, comparable to the level of the PMMA filling, was chosen to be H_g_ = 80 nm. The proposed filters are accordingly expected to exhibit suppressed transmissions centered at λ = 420 nm, 532 nm, and 600 nm, respectively. Taking into account that the subtractive coloration delivers a photon throughput twice as much as that of the additive coloration pertaining to red, green, and blue, the structural color filter is preferred to deliver an enhanced transmission leading to a strong color signal^22, 23^. Toward that end, an ultra-thin a-Si:H film of 80-nm thickness was deposited on a glass substrate via plasma-enhanced CVD process of SiH_4_ (silane) at 250 °C. The layer was subsequently plasma-etched via electron-beam patterning so as to produce a 2D array of a-Si:H nanodisks. PMMA was finally spin-coated to cover the gap between the nanodisks. Scanning electron microscope (SEM) images of the fabricated metasurface for the colors of yellow, magenta, and cyan are presented in Fig. 2(a) from left to right, revealing sophisticatedly defined a-Si:H nanodisks. As revealed in the insets, the PMMA layer has been filled up to the height of the nanodisk. For the proposed device, the calculated and measured transmission spectra in the visible band are plotted in Fig. 2(b). The dips that correspond to a drastically reduced transmission are observed at λ = 420 nm, 528 nm, and 595 nm. It is noted that for the case of the yellow filter, the transmission dip observed at the wavelength of ~420 nm is relatively shallow. The corresponding bandwidth is relatively broad since the resonance mediated by the Mie scattering is suppressed due to the higher loss in a-Si:H in the shorter wavelengths compared to that in longer ones. The influence of absorption in the shorter wavelength regime will be discussed later. The filter devices were observed to provide high transmission efficiencies surpassing 90% in the off-resonance region longer than their resonant wavelengths. The images of the bright yellow, magenta, and cyan color outputs that emerge from the fabricated devices, assuming a footprint of 40 μm × 40 μm, were taken with the help of a digital microscope (Leica DM4000 M). As illustrated in Fig. 2(c), the chromaticity coordinates for the three primary colors are mapped in the CIE 1931 diagram, indicating that the measurement is in good correlations with the calculation.

### Tailoring the color output through the adjustment of structural parameters

**Figure 3:**
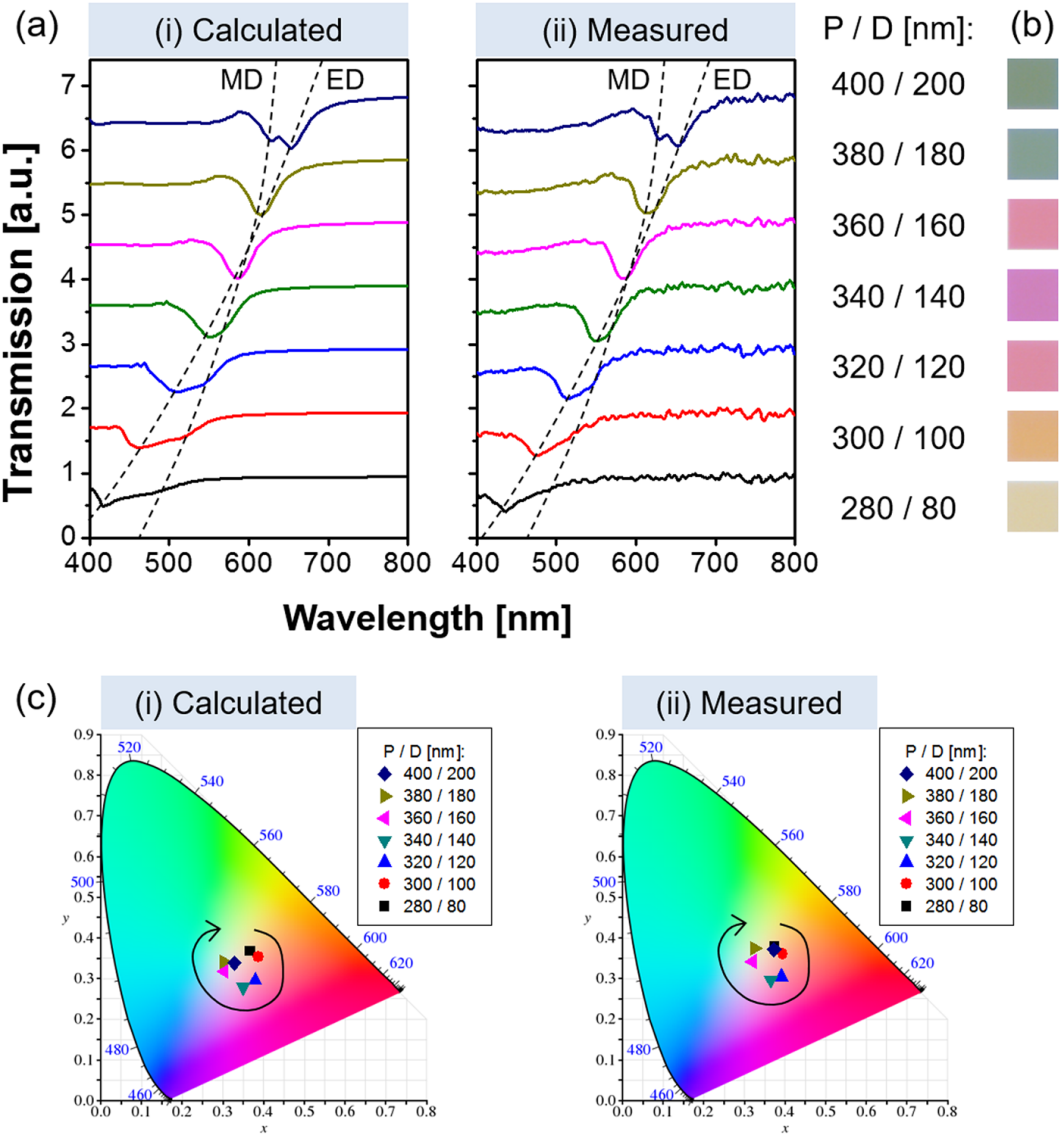
(**a**) (i) Calculated and (ii) measured transmission spectra when the period is varied from P = 280 nm to 400 nm under the relationship of P = D + 200 [nm], while the diameter is varied from D = 80 nm to 200 nm. The transmission dips for the ED and MD are traced by the dashed line. (**b**) Corresponding color images that are captured by an optical microscope. (**c**) Chromaticity coordinates corresponding to (i) the calculated and (ii) the measured spectra in the CIE 1931 diagram.

Regarding a structural color filter, the manipulation of color can be carried out via the structural parameters. In the case of a metasurface that exploits cylindrical nanoparticles, the resonance condition is controlled by modifying the height and diameter^8^. In an effort to pursue a broad color response, the transmission spectra were inspected in terms of the period and diameter of a nanodisk over the entire visible band. For the a-Si nanodisk, the period was varied from 280 nm to 400 nm in steps of 20 nm in accordance with the relationship of P = D + 200 [nm], when the diameter was scanned from 80 nm to 200 nm. The effect of the height H_g_ on the spectra is described in supplementary Fig. S2 ^9, 10^. Though the location of the transmission dips can be altered by tuning the height, it was fixed at 80 nm to serve an appropriate spectral response and corresponding color output. As plotted in Fig. 3, various colors were obtained hinging on the period and diameter. The transmission spectra are depicted in detail in Fig. 3(a). Figure 3(a)(i) shows the evolution of the spectral response, resulting in a broad range of resonance that runs from 415 nm to 626 nm. The measured spectra for the seven color filters of concern are plotted in Fig. 3(a)(ii), yielding a good agreement with the calculated results. Double transmission dips appeared for a color filter with P = 400 nm and D = 200 nm. Meanwhile, the device that produces a yellowish color rarely exhibited such dips, which may be imputed to the light absorption that is incurred by the extinction of the a-Si:H material. A metasurface utilizing c-Si nanodisks, which is known to have lower extinction than the case of a-Si:H, was taken into account to scrutinize the spectral characteristics. As in the case of the proposed structure in a-Si:H, it was assumed that the substrate is made of glass when the gap between c-Si nanodisks is filled with PMMA. The values of P and D were similarly scanned in steps of 20 nm. The indices of refraction of c-Si as available from Palik are depicted in Fig. S1 ^41^. As shown in Fig. S3(a), two resonance bands were apparently observed for the device having smaller values of P and D, whose resonance is predicted to belong to the shorter wavelength region. Noting that the ED resonance results in a much larger wavelength shift in response to the nanodisk diameter compared with the case of the MD resonance^8^, the locations of the transmission dips that stem from the ED and MD resonances are individually estimated and traced by a dashed line, respectively. As shown in Fig. 3(b), the obtained colors vary from light yellow through magenta to cyan and green. The relevant calculated and measured chromaticity coordinates are plotted in the CIE 1931 diagram in Fig. 3(c)(i) and (c)(ii), respectively, where the evolution of the color output is marked by a black arrow. Consequently, it was confirmed that the proposed metasurface that exploits an a-Si:H nanodisk efficiently renders a broad color palette, whereas for the c-Si metasurface the color reproduction is unsatisfactory, as described in Fig. S3(b).

### Investigation of the resonances underlying the transmission dips for the a-Si:H nanodisk

**Figure 4:**
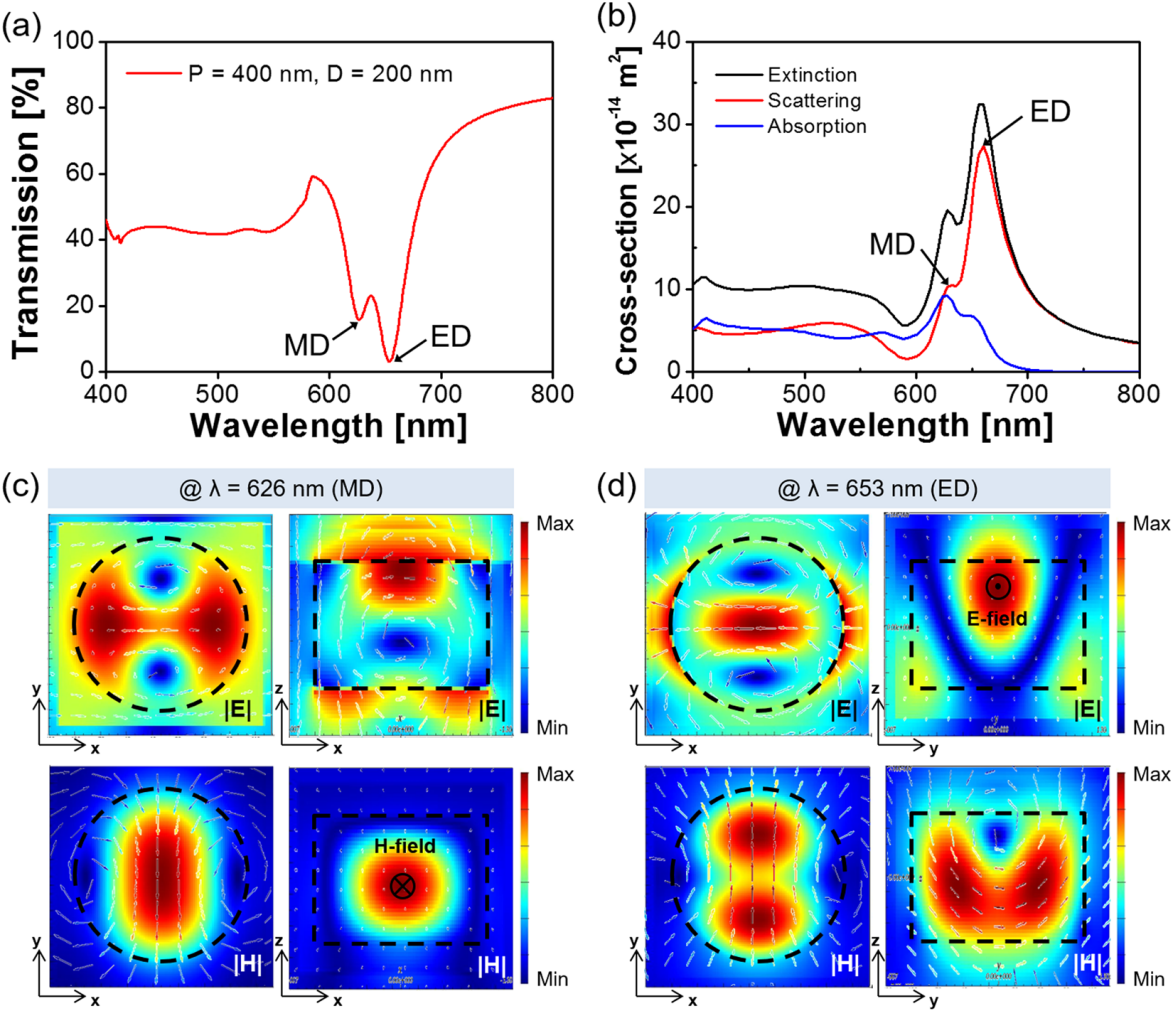
(**a**) Calculated transmission spectra for the metasurface with P = 400 nm and D = 200 nm, providing two resonances at λ = 626 nm and 653 nm. (**b**) Calculated extinction, scattering, and absorption cross-sections for the a-Si:H nanodisk. Field profiles at (**c**) λ = 626 nm and (**d**) λ = 653 nm indicating enhanced H- and E-field in the middle of the nanodisk, indicating a strong MD and ED resonance, respectively.

**Figure 5:**
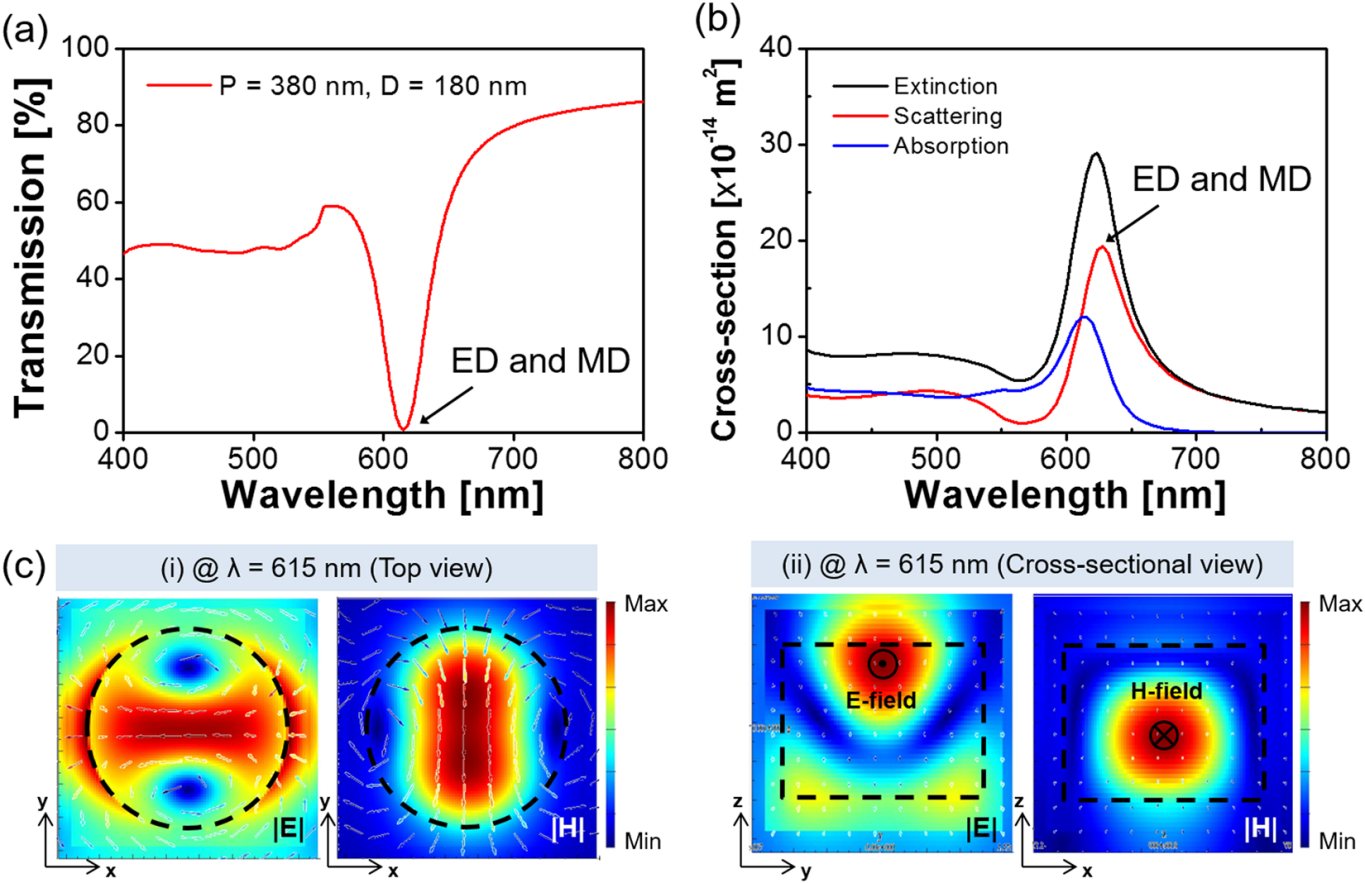
(**a**) Calculated transmission spectra for the metasurface with P = 380 nm and D = 180 nm, exhibiting a single resonance at λ = 615 nm. (**b**) Calculated extinction, scattering, and absorption cross-sections for the a-Si:H nanodisk. (**c**) Resonant field profiles for the unit a-Si:H nanodisk at λ = 615 nm: (i) top view and (ii) cross-sectional view. Both the ED and MD resonances coincide spectrally.

**Figure 6:**
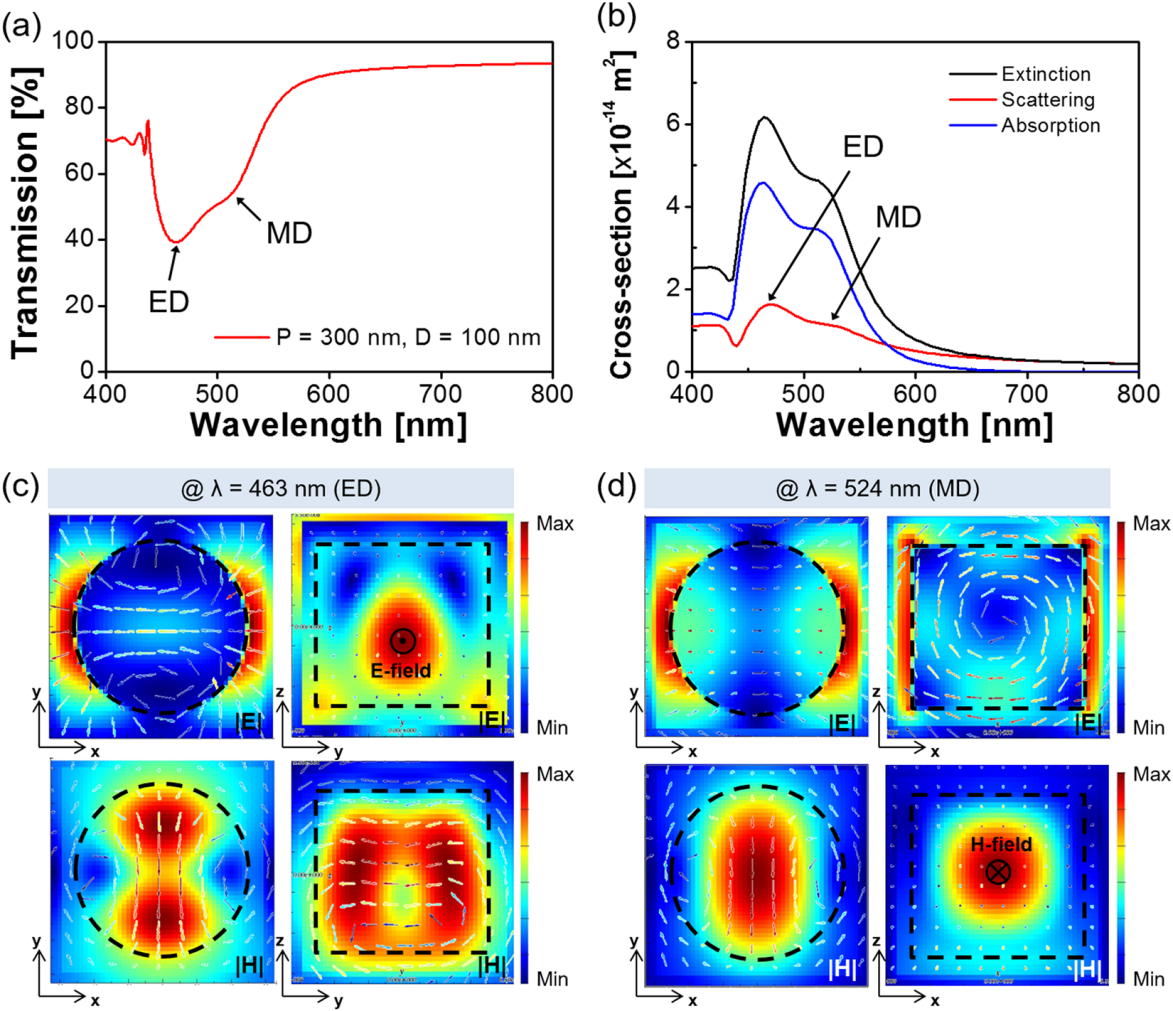
(**a**) Calculated transmission spectra for the metasurface with P = 300 nm and D = 100 nm, providing double resonances at λ = 463 nm and 524 nm. (**b**) Calculated extinction, scattering, and absorption cross-sections for the a-Si:H nanodisk. Field profiles for (**c**) the ED resonance at λ = 463 nm and (**d**) the MD resonance at λ = 524 nm.

As for the nanoparticles that involve high-index materials like Si, the ED and MD resonances are supposed to be mostly induced via the Mie scattering^7–11^. To explore the operational principle of the a-Si:H metasurface, the constituting nanodisks were rigorously assessed in terms of the scattering cross-section in conjunction with the field profile. A periodic boundary condition was adopted to address the coupling between adjacent nanodisks^42^. Three different devices were theoretically analyzed through the FDTD simulations, when the observation planes for the field profiles are depicted in Fig. 1(b). For the filter with P = 400 nm and D = 200 nm, as shown in Fig. 4(a), the transmission exhibited two separate dips that are positioned at λ = 626 nm and 653 nm. In order to expound the double resonances, the extinction, absorption, and scattering cross-sections were accordingly investigated as shown in Fig. 4(b). It is reckoned that the spectral transmission is dominantly governed by the scattering. The field profiles in relation to the dipole resonances, which are initiated by the scattering, are described in Fig. 4(c) and (d). The MD, which occurs at λ = 626 nm in the shorter wavelength region, is signified by the reinforced H-field that is peaked in the middle, in combination with the enhanced circular E-field. Meanwhile, the ED resonance, which is found at the longer wavelength of 653 nm, can be attested by the strengthened E-field that develops near the center of the disk, as shown in Fig. 4(d). Both the ED and MD resonances were categorically proved in view of the observed field profiles. For another device with P = 380 nm and D = 180 nm, a single resonance was observed for the transmission that is given in Fig. 5(a), while the scattering was predicted to bring about a single peak at λ = 615 nm in view of the calculated cross-section that is plotted in Fig. 5(b). The ED and MD resonances are monitored to nearly overlap under a fixed period or diameter^8^. As shown in Fig. 5(c)(ii), the cross-sectional view for the resonance represents the concurrent enhancement of the E- and H-field around the center of the nanodisk. We finally elaborated on the Mie scattering-induced resonance for a metasurface with P = 300 nm and D = 100 nm, recognizing that the a-Si:H material is particularly subject to a relatively high extinction below λ = 550 nm. The spectra in Fig. 6(a) exhibit no distinct transmission dip, unlike the previous cases adopting larger values of P and D. In light of the extinction cross-section that is presented in Fig. 6(b), the absorption is judged to dictate the scattering. The field profiles for λ = 463 nm and 524 nm, which are respectively shown in Fig. 6(c) and (d), suggest no remarkable field enhancement in the nanodisk, in comparison with the case of a c-Si nanodisk. For the c-Si metasurface with P = 300 nm and D = 100 nm, the calculation results are supplied in supplementary Fig. S4. Figure S4(a) and (b) signify that the metasurface assumes a near-zero transmission dip in conjunction with an elevated scattering cross-section, which is comparable to the absorption cross-section. In view of Fig. S4(c) and (d), the metasurface that uses a c-Si nanodisk of a lower extinction can lead to a higher field enhancement than the case of a-Si:H. It is hence concluded that the absence of an obvious transmission dip in the shorter wavelength region is primarily ascribed to a diminished amount of scattering. Despite the absorption pertaining to the a-Si:H nanodisk, the identities of the ED and MD resonance can be discovered by virtue of the field profiles.

## Discussion

Considering that Si is perceived as a viable platform for optical devices in view of its low optical loss in the visible spectral range, we created a dielectric metasurface that consists of an a-Si:H nanodisk and applied it to develop highly efficient subtractive colorations. The achieved transmission efficiency exceeded 90%, providing high-fidelity fabrication to the design. Although it is extremely challenging to grow a high quality of c-Si on a foreign substrate, a-Si:H was adopted as a prominent alternative to guarantee a cost-effective fabrication scheme that is empowered by the CMOS process. The ED and MD resonances, which transpire even under the predominant absorption of a-Si:H, could be recognized by examining the scattering cross-section alongside the field profiles. It was categorically verified that the a-Si:H material could be prepared to provide salient features in terms of the high productivity, simple fabrication process, and high fidelity. Although a-Si:H is a little lossy in the short visible band, the metasurface based on a-Si:H is expected to be an affordable alternative to the plasmonic color filter, in light of the achieved high transmission in conjunction with the cost-effective, facile fabrication. The spin-cast polymeric layer was useful for facilitating the planarization that is crucial for the practical integration of image sensors^33, 34^ and stretchable filters^43^. The proposed filters will be readily applicable for the implementation of color displays, imaging devices, photovoltaics, and photorealistic color printing.

## Methods

### Numerical simulation

The refractive index of the a-Si:H film, which was checked using a reflecto-spectrometer (Filmtek4000, SCI) that operates in the spectral range from 450 nm to 1650 nm, was reflected to the simulations. The index of c-Si was obtained from Palik^41^, while the properties of the other materials were derived from the multi-coefficient model offered by the simulation tool (FDTD Solutions, Lumerical, Canada)^44^. For the proposed dielectric metasurface, the transmission spectra, extinction cross-sections, and field profiles were investigated by means of the FDTD method-based tool. A normally incident plane wave was illuminated to a unit cell that satisfies a periodic boundary condition, so that an array of periodically arranged Si nanodisks could be emulated.

### Device fabrication

The proposed color filters were designed and manufactured to exhibit dimensions of 40 μm × 40 μm. An 80-nm thick a-Si:H film was deposited on a glass substrate by virtue of a gas mixture of SiH_4_ (silane) and helium carrier via plasma enhanced chemical vapor deposition (PECVD) (Oxford, Plasmalab System 100). The layer was subsequently patterned through an electron-beam lithography system (RAITH 150), which adopted a positive resist of ZEP520A, then dry etched with a plasma etcher (Oxford Plasmalab System 100) using a gas mixture of CHF_3_ and SF_6_. A polymeric PMMA film was finally spin coated on top of the patterned a-Si:H layer and cured so as to fill up the region in the vicinity of the produced nanodisks.

### Optical characterization

The completed a-Si:H pattern was visually evaluated under a high-resolution field emission scanning electron microscope (FESEM S-4800, Hitachi). The transmission spectra were examined for different polarizations by launching a collimated beam via a multimode fiber, which originates from a halogen lamp (HL-2000-FHSA, Ocean Optics) that is properly polarized via a calcite crystal polarizer (GTH 10M-A, Thorlabs), to the prepared filter that is mounted on a motorized rotation stage via a focusing lens. The optical output was captured by a spectrometer (Avaspec-3648, Avantes). The images relating to each pixel of the color filter were taken by a digital microscope (Leica DM4000 M).

## Electronic supplementary material

Supplementary Information
