# Supplementary material for: Structural Color Filters Enabled by a Dielectric Metasurface Incorporating Hydrogenated Amorphous Silicon Nanodisks
Source: Sci Rep. 2017 May 31;7:2556. doi: 10.1038/s41598-017-02911-w (PMC5451390; doi:10.1038/s41598-017-02911-w)
Supplement: Supplementary file 1 — Supplementary Information [file 41598_2017_2911_MOESM1_ESM.pdf]

SUPPLEMENTARY INFORMATION

Structural Color Filters Enabled by a Dielectric Metasurface Incorporating Hydrogenated Amorphous Silicon Nanodisks

Chul-Soon Park[1,], Vivek Raj Shrestha[1,2,*], Wenjing Yue[1], Song Gao[1], Sang-Shin Lee[1], Eun-Soo Kim[1], and Duk-Yong Choi[3]*

[1]Department of Electronic Engineering, Kwangwoon University, 20 Kwangwoon-ro, Nowon-gu, Seoul 01897, South Korea

[2]School of Physics, The University of Melbourne, Melbourne, Victoria 3010, Australia

[3]Laser Physics Centre, Research School of Physics and Engineering, Australian National University, Canberra ACT 2601, Australia

[*]*These authors contributed equally to this work.*

Correspondence and requests for materials should be addressed to S.S.L. (email: slee@kw.ac.kr) and D.Y.C. (email: duk.choi@anu.edu.au*)*

Keywords: Optical materials and structures, Metamaterials, Nanophotonics and plasmonics

1. Material properties used for the calculation.

Figure S1 shows the refractive indices of a-Si:H and c-Si. The former was obtained by characterizing a film that is deposited on a glass substrate with the help of a reflecto-spectrometer, while the latter is available from Palik [1].

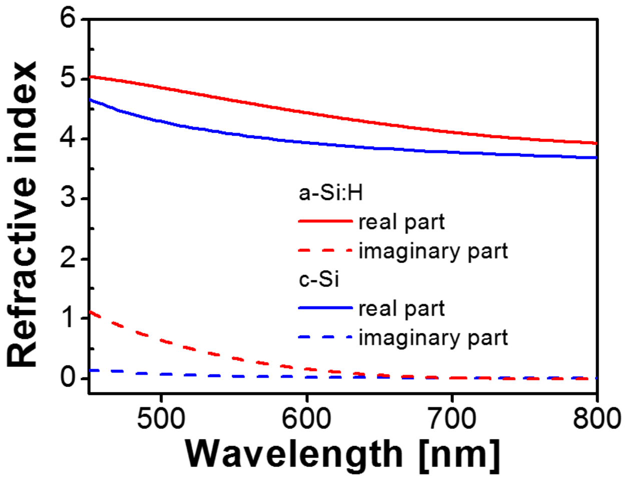

Supplementary Figure S1. Refractive indices of a-Si:H and c-Si that are used for the calculation.

2. Effect of the height (H_g) of a-Si:H nanodisks on the device performance.

The location of the transmission dips pertaining to the electric dipole (ED) and the magnetic dipole (MD) can be altered by changing the height (H_g). As H_g is increased, both the ED and MD resonance dips tend to be red-shifted, with the MD resonance occurring in the longer wavelength region than the ED resonance. For H_g beyond 100 nm, it is particularly noted that small transmission dips, which are accounted for by a multi-pole resonance such as a magnetic quadrupole (MQ), are observed to manifest in the spectral band shorter than the ED and MD resonances [2, 3], as indicated in Figure S2.

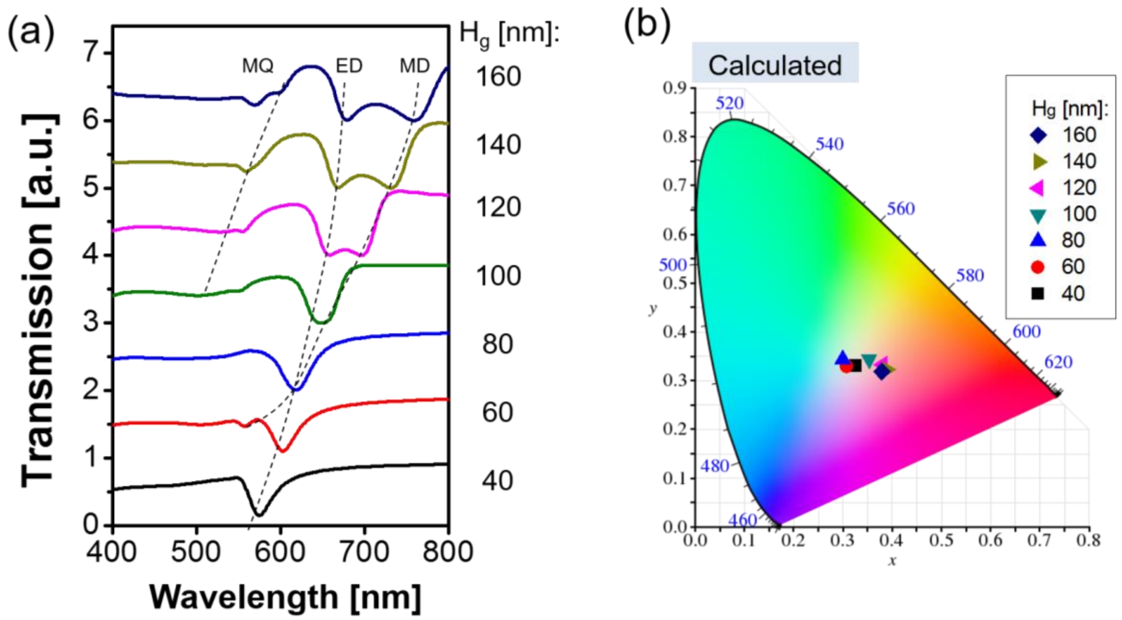

Supplementary Figure S2. (a) Calculated transmission spectra for the metasurface with P = 380 nm and D = 180 nm, while H_g is varied from 40 nm to 160 nm. (b) CIE 1931 chromaticity diagram for the corresponding color output.

3. Performance of the metasurface based on a c-Si nanodisk.

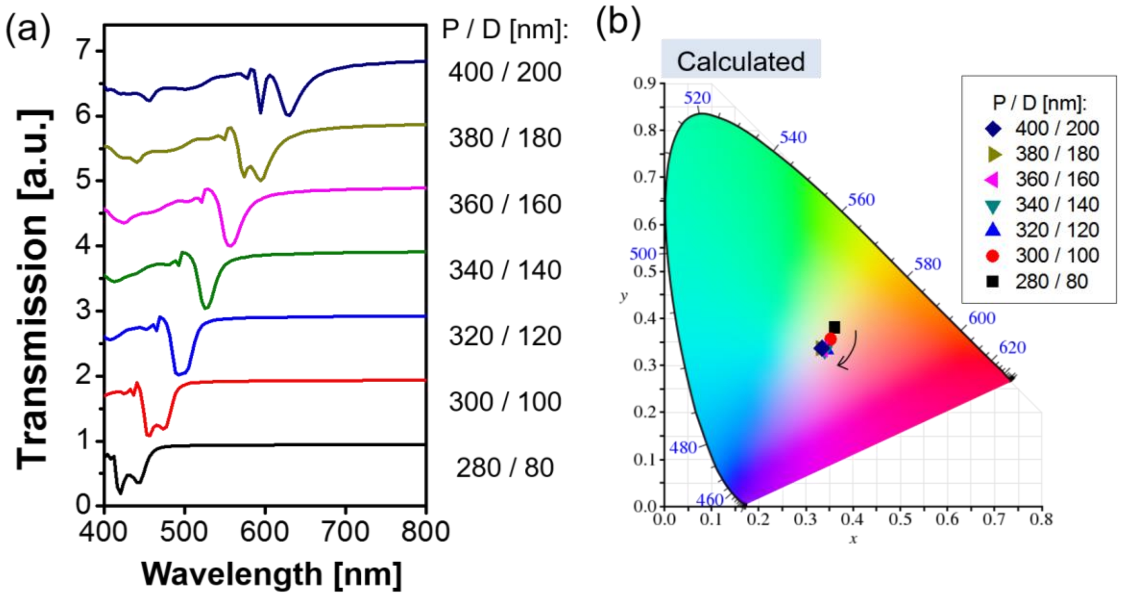

Supplementary Figure S3. (a) Calculated transmission spectra for the metasurface exploiting a c-Si nanodisk when the period is varied from P = 280 nm to 400 nm under the relationship of P = D + 200 [nm], while the diameter is varied from D = 80 nm to 200 nm. (b) CIE 1931 chromaticity diagram for the corresponding color output.

4. Observation of the Mie scattering-induced resonance for the metasurface that engages a c-Si nanodisk.

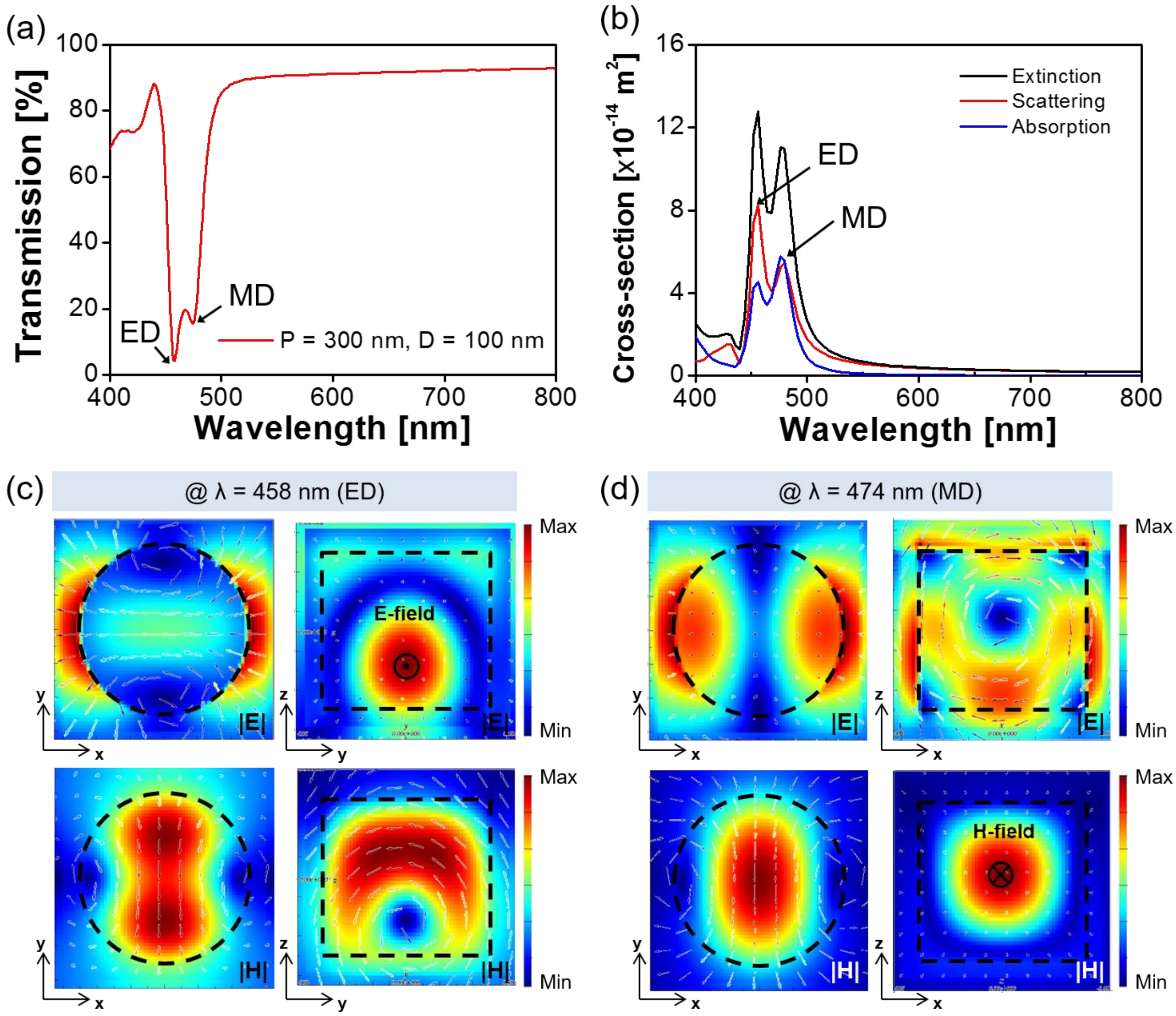

Supplementary Figure S4. (a) Calculated transmission spectra for the metasurface with P = 300 nm and D = 100 nm, rendering two distinct resonances at λ = 458 nm and 474 nm. (b) Calculated extinction, scattering, and absorption cross-section for the c-Si nanodisk. Field profiles for (c) the ED resonance at λ = 458 nm and (d) the MD resonance at λ = 474 nm.

References

[1] E. D. Palik, *"Handbook of Optical Constants of Solids III,"* (Academic Press, San Diego, USA, 1998).

[2] A. I. Kuznetsov, A. E. Microshnichenko, Y. H. Fu, J. B. Zhang, and B. Luk'yanchuk, "Magnetic light," *Sci. Rep.* **2,** 492 (2012).

[3] J. van de Groep and A. Polman, "Designing dielectric resonators on substrates: Combining magnetic and electric resonances," *Opt. Express* **21,** 26285-26302 (2013).
